# Novel treatment combining antiviral and neutralizing antibody-based therapies with monitoring of spike-specific antibody and viral load for immunocompromised patients with persistent COVID-19 infection

**DOI:** 10.1186/s40164-022-00307-9

**Published:** 2022-09-09

**Authors:** Daiki Wada, Yasushi Nakamori, Shuhei Maruyama, Haruka Shimazu, Fukuki Saito, Kazuhisa Yoshiya, Yasuyuki Kuwagata

**Affiliations:** 1 Department of Emergency and Critical Care Medicine, Kansai Medical University General Medical Center, 10-15 Fumizono-cho, Moriguchi, Osaka 570-8507 Japan; 2 Department of Emergency and Critical Care Medicine, Kansai Medical University Hospital, 2- 3-1 Shinmachi, Hirakata, Osaka 573-1191 Japan

**Keywords:** COVID-19, SARS-CoV-2

## Abstract

Because prolonged viral replication of SARS-CoV-2 is increasingly being recognized among immunocompromised patients, subacute or chronic COVID-19 pneumonia can cause persistent lung damage and may lead to viral escape phenomena. Highly efficacious antiviral therapies in immunosuppressed hosts with COVID-19 are urgently needed. From February 2022, we introduced novel treatment combining antiviral therapies and neutralizing antibodies with frequent monitoring of spike-specific antibody and RT-PCR cycle threshold (Ct) values as indicators of viral load for immunocompromised patients with persistent COVID-19 infection. We applied this treatment to 10 immunosuppressed patients with COVID-19, and all completed treatment without relapse of infection. This may be a potentially successful treatment strategy that enables us to sustain viral clearance, determine optimal timing to stop treatment, and prevent virus reactivation in immunocompromised patients with persistent COVID-19.

To the Editor,

The coronavirus disease 2019 (COVID-19) pandemic has caused significant morbidity and mortality worldwide. Driven by findings of high-quality randomized trials, management of patients with severe acute respiratory syndrome coronavirus 2 (SARS-CoV-2) infection has developed rapidly over the past year [1]. Although these trials justifiably focused on preventing severe disease in general patients, their benefits might not apply to some immunodeficient patients at high risk for recurrence of persistent infection. Prolonged Covid-19 is a developing issue for patients with lymphoma or immune deficiency [2]. Several reports described persistent SARS-CoV-2 replication with severe symptoms in immunocompromised patients, including those with lymphoma [3, 4]. This population is at increased risk of persistent SARS-CoV-2 infection, severe outcomes, and mortality due to COVID-19 [5]. Host genomic evolution and viral escape phenomena may potentially occur in patients with primary immunodeficiency due to prolonged relapse of SARS-CoV-2-related infection [6]. Clark et al. reported that SARS-CoV-2 evolution in an immunocompromised host shows neutralization escape mechanisms [7]. As an underlying defect in the immune response of patients with haematological malignancies, the lack of B-cell precursors is the main reason for continuing viral replication and defective viral clearance [8]. Furthermore, immune system deficiencies occurring following anti-CD20 monoclonal antibody treatment can slow development of neutralizing antibodies after administration of two doses of mRNA vaccines against SARS-CoV-2 [9]. Thus, highly efficacious antiviral therapies are urgently needed for these patients. Furlan et al. noted that therapeutic strategies combining immunotherapy with prolonged antiviral treatment may be decisive in patients with B cell immunodeficiencies [10]. Although rapid viral elimination with combined antiviral and antibody-based therapy might preclude further evolution, no optimal, decisive strategy is currently available for patients with persistent infection that allows clinicians to sustain viral clearance, determine optimal timing to stop treatment, and prevent virus reactivation. Some reports discuss RT-PCR cycle threshold (Ct) values and specific antibodies that indicate prolonged COVID-19 infection and responses to vaccines, but none address use of both indicators for treatment [11, 12].

From February 2022, we introduced a novel treatment combining antiviral and neutralizing antibody-based therapies with monitoring of spike-specific antibody and Ct values as indicators of viral load for immunocompromised patients with persistent COVID-19 infection. Knowledge of specific immune responses to antibody-based therapy in immunosuppressed patients is important, and well-validated quantitative PCR that correlates with both viral culture titres and Ct values may help in clarifying infectious viral shedding, guiding treatments, and assessing outcomes [11]. We examined these titre values at least twice weekly. Monitoring of spike-specific antibody response and Ct values during treatment allowed us to evaluate effects of antivirals and neutralizing antibody-based therapies and determine when to end treatment.

In an immunosuppressed COVID-19 patient taking tacrolimus hydrate and mycophenolate mofetil following kidney transplantation, we administered remdesivir as antiviral therapy on day 1 along with sotrovimab as neutralizing antibody-based therapy (Fig. 1). We continued remdesivir while monitoring spike-specific antibody response, viral load, and Ct values. Because the latter two values did not improve, we changed to nirmatrelvir/ritonavir antiviral therapy, following which these values improved to treatment level. After the patient’s viral load stopped rising without antiviral therapy and antibody response was maintained, the patient was removed from isolation and discharged on day 23. We applied this treatment strategy to 10 immunosuppressed COVID-19 patients who currently or previously received immunosuppressive agents for their disease (Table 1). In 5 patients, we switched from initial remdesivir to other antiviral therapy during treatment because viral load and Ct values remained unchanged or worsened. In the other 5 patients who responded well to remdesivir, we terminated it only after confirming that viral load and Ct values reached target levels. All patients were removed from isolation after confirmation that viral load, Ct values, and antibody titres had not worsened since end of treatment. No patient suffered relapse of the viral infection.

**Table 1:** Immunosuppressed patients with COVID-19 completing treatment without relapse of the viral infection

Factors	Patient identification									
	**1**	**2**	**3**	**4**	**5**	**6**	**7**	**8**	**9**	**10**
Age (years)	51	74	49	94	51	66	72	57	51	84
Sex (male/female)	M	M	F	F	F	F	M	M	M	M
Primary disease	Follicular lymphoma	Follicular lymphoma	Myasthenia gravis	Myasthenia gravis Rheumatoid arthritis	Kidney transplant	Kidney transplant	Kidney transplant	Liver transplant	Chronic myeloid leukemia	Chronic lymphocytic leukemia
Other comorbidity	None	Hypertension	Thyrotoxicosis	Hypertension	Epilepsy	Hypertension	Hypertension Diabetes mellitus	Hypertension Diabetes mellitus Nephrotic syndrome	None	Diabetes mellitus Coronary heart disease
COVID-19 vaccination	Yes	Yes	No	Yes	No	Yes	Yes	Yes	No	Yes
Anti-CD20 antibody	Obinutuzumab	Rituximab Obinutuzumab	None	None	None	None	None	None	None	None
Immunosuppressive agents for primary disease	C**y**clophosphamide Prednisolone	C**y**clophosphamide Prednisolone	Tacrolimus	Tacrolimus Prednisolone	Tacrolimus Mycophenolate mofetil Everolimus Prednisolone	Tacrolimus Mycophenolate mofetil Methylprednisolone	Tacrolimus Mycophenolate mofetil Methylprednisolone	Tacrolimus Mycophenolate mofetil Everolimus	Iguratimod Tocilizumab Prednisolone	Prednisolone
Initial antiviral therapy Switched antiviral therapy	Remdesivir	Remdesivir Nirmatrelvir/Ritonavir	Remdesivir Nirmatrelvir/Ritonavir	Remdesivir Molnupiravir	Remdesivir	Remdesivir Nirmatrelvir/Ritonavir	Remdesivir	Remdesivir Nirmatrelvir/Ritonavir	Remdesivir	Remdesivir
Neutralizing antibody-based therapy	Sotrovimab Casirivimab/Imdevimab	Sotrovimab	Casirivimab/Imdevimab	Casirivimab/Imdevimab	Sotrovimab	Sotrovimab	Sotrovimab	Sotrovimab	Casirivimab/Imdevimab	Sotrovimab
Initial Ct value	18.6	19.8	25.3	18.2	19.6	25	17.9	22.4	15	25.6
Initial spike-specific antibody	Negative	Negative	163 U/mL	Negative	Negative	Negative	Negative	Negative	Negative	1.41 U/mL
Length of antiviral and antibody-based therapy	10 days	14 days	9 days	10 days	10 days	21 days	20 days	18 days	8 days	7 days
Length of hospital stay	14 days	19 days	13 days	28 days	13 days	23 days	43 days	18 days	12 days	17 days

As limitations, first, although the progression of SARS-CoV-2 infectious viral shedding and specific immune responses by this treatment are understandable, criteria for frequency of measuring spike-specific antibody and Ct values remain unknown. Second, long-term data on potential viral relapse is unknown. Third, administration of antiviral drugs at above-normal prescription limits based on frequent test results could increase long-term drug and hospitalization costs. Fourth, because the sample size is small, results should be interpreted cautiously. More rigorous research including randomization and larger sample size is needed. Nevertheless, our novel strategy may offer potentially successful treatment for immunocompromised patients with persistent COVID-19.

**Fig. 1:**
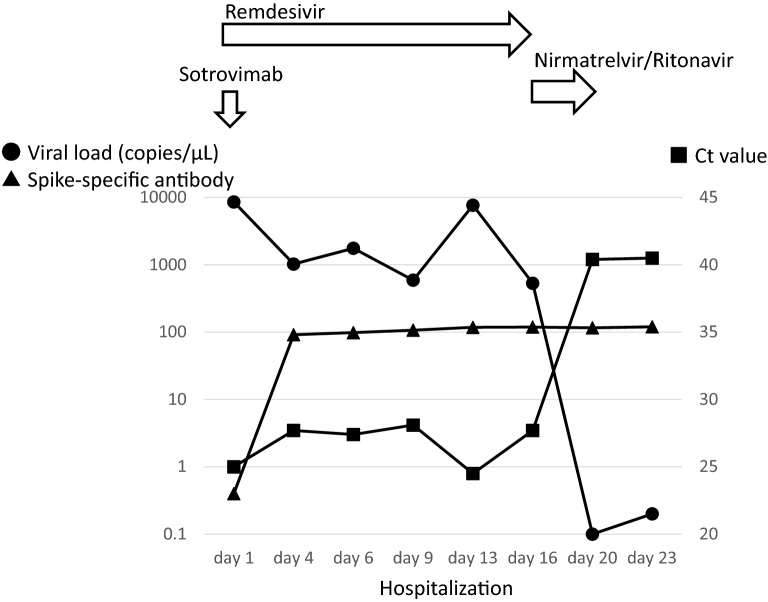
The graph shows the clinical course of a COVID-19 patient taking tacrolimus hydrate and mycophenolate mofetil as immunosuppressive therapy following kidney transplantation. The round circle indicates viral load, the square indicates Ct value, and the triangle indicates the spike-specific antibody

## Data Availability

This case report only contains clinical data from the medical records of the patient reported herein. The data will be made available upon request.

## Abbreviations

COVID-19: Coronavirus disease 2019
SARS-CoV-2: Severe acute respiratory syndrome coronavirus 2
Ct: RT-PCR cycle threshold
